# Cerebrospinal fluid in multiple sclerosis

**DOI:** 10.4103/0972-2327.58282

**Published:** 2009

**Authors:** Kottil W. Rammohan

**Affiliations:** Clinical and Experimental Neuroimmunology, Department of Neurology, The Ohio State University, Columbus, Ohio 43221 USA

**Keywords:** Cerebrospinal fluid, multiple sclerosis

## Abstract

**Background:**

Technological advances have made it possible to examine the human cerebrospinal fluid (CSF) in a manner that was previously impossible. CSF provides a window into the changes that occur in the central nervous system (CNS) in health and disease. Through analysis of the CSF, we discern indirectly the state of health of the CNS, and correctly or incorrectly, draw conclusions regarding mechanisms of CNS injury and repair.

**Objective, Materials and Methods:**

To review the current state of knowledge of changes in the CSF in multiple sclerosis.

**Discussion:**

Establishing CSF markers that permit evaluation of the various biological processes in multiple sclerosis remains a challenge. Of all the biological processes, inflammatory markers are probably the best identified. Detection of oligoclonal immunoglobulin bands in the CSF is now established as the single most useful laboratory marker in the CSF to aid in the diagnosis of multiple sclerosis. Markers of demyelination, remyelination, neuro-axonal loss, neural repair and regeneration, and astrogliosis are only now being recognized. A good surrogate for any of these pathophysiological processes has not been defined to date.

**Conclusion:**

The goal of future research is not only to define surrogate markers in the CSF for each of the above functions, but also to extend it to other more readily accessible body fluids like blood and urine. A synopsis of the current literature in most of these areas of CSF evaluation pertaining to multiple sclerosis is presented in this article.

The cerebrospinal fluid (CSF) has been the focus of attention in multiple sclerosis for a very long time. The “colloidal gold curve” was used in the diagnosis of multiple sclerosis (MS) before the advent of modern techniques of protein separation and evaluation.[1–2] A “paretic” pattern (as in “general paresis” in syphilis of the central nervous system) or “first zone” elevation in this assay was considered corroborative of being diagnostic of this disorder. The basis of changes seen in the colloidal gold curve assay are not known but the first zone pattern described in this assay was probably a reflection of the presence of immunoglobulins in the CSF commonly seen in MS as well as in general paresis of syphilis.

The CSF is clear and colorless in all patients with MS, and most patients have normal cell counts and total protein levels. Even during an acute exacerbation, total CSF protein and cell counts remain normal, although sometimes a modest mononuclear pleocytosis can be identified. Protein levels of over 100 mg/dL are distinctly unusual in MS and should alert the physician to an alternate diagnosis as also pleocytosis of over 100 cell mm^3^. What has become clear over the years is the fact that CSF IgG levels or IgG index are consistently elevated, 24 h intrathecal IgG synthesis is abnormally increased, and the IgG produced in the CNS has a restricted charge pattern, resulting in an abnormal electrophoretic profile known as oligoclonal bands (OCBs). In addition to typical large and abundant proteins like prealbumin, albumin, transferrin, and immunoglobulins that can be identified by standard electrophoresis, many other proteins have been identified in the CSF of MS patients by using advanced sensitive techniques. Today, there are > 400 proteins that have been detected in normal CSF, and some of these proteins show promise as markers for the disease process when expressed in abnormal amounts in the CSF. Additionally, investigation has extended changes in the CSF to lipids and nucleic acids. The goal of this communication is to provide an overview of most of the recent advances in our understanding of changes in the CSF in MS. The reader is referred to reviews on specific topics for additional information, as an in-depth discussion on all these topics is beyond the scope of this limited review.

## MS and Oligoclonal bands

Abnormal elevated intrathecal IgG synthesis is the basis of the OCBs in MS. The elevated *IgG Index*, also known as the “Link Index”, was defined by Hans Link and colleagues as the ratio of CSF IgG to CSF albumin to the ratio of serum IgG to serum albumin.[3,4] This ratio-of-a-ratio when greater than 0.7 (or the defined value for the laboratory), was indicative of intrathecal synthesis of IgG. Tourtellotte and colleagues established a formula for the determination of intrathecal IgG synthesis for a 24 hour period and values in excess of 4 mg per 24 h period (or values established by the laboratory) were considered abnormal.[5,6] Although these quantitative measures of intrathecal IgG were helpful, the most useful test in the CSF of MS patients was demonstration of the OCBs. In 1942, Elvin Kabat described elevated “gamma globulin” in CSF from patients with MS for the first time.[7] While at Columbia University, New York, he described using the then novel technique of agarose gel electrophoresis to show abnormal elevation of gamma globulins but not albumin or transferrin in the CSF from patients with multiple sclerosis, but not from control subjects. Subsequently in 1957 in Antwerp, Belgium, Denise Karcher and colleagues in Armand Lowenthal's laboratory first described the presence of oligoclonal bands in the CSF of MS patients by using agarose gel electrophoresis.[8] They described the detection of oligoclonal bands in unconcentrated CSF using a silver staining method[9] that was a modification of the techniques of Kerenyi and Galyas for agarose gels.[10] The initial technique of silver staining that they had developed was cumbersome and analysis of a single CSF sample required as many as three days. Subsequently, we modified this technique and adapted it to permit simultaneous analysis of as many as 20 CSF samples in three hours.[11] As silver staining techniques are cumbersome, we developed and introduced in 1986 the immunoblot analysis of unconcentrated CSF, which permitted the detection of OCBs in unconcentrated CSF.[12] Silver enhancement of 3',4'-diaminobenzidine (DAB) further allowed the dilution of normal CSF by ten fold if necessary, and this technique also permitted the examination of microliter quantities of CSF from mice and the analysis of other body fluids like tears for OCBs.[13]

After developing the immunoblot analysis of CSF in 1986, we have routinely employed this technique at our institution for examination of CSF for OCBs in over 10,000 samples (∼ 600 samples per year). The silver staining techniques for agarose gels and the current technique of immunoblot analysis of CSF are described below:

## Silver staining of agarose gels

Agarose isoelectric focusing was carried out by using standard techniques. Briefly, isoelectric focusing of unconcentrated CSF was carried out at constant current using 1% zero-EEO agarose containing 10% ampholines, pH 3.5 to 10. At completion of the procedure, the gel was fixed in 60% methanol and 4% acetic acid, washed liberally in deionized water, dried using a hair dryer, and stained using silver as described below.

All reagents used for staining are described in Table 1. A 2% (w/v) solution of potassium ferrocyanide was prepared in acetate buffer, pH 6, and a sufficient quantity was used to soak the dried gel. After 10 minutes, the gel was washed with copious amounts of deionized water, three times for 5 minutes each, for a total wash time of 15 minutes. Equal volumes of solutions A and B [Table 1] were mixed by a slow dropwise addition of solution B to solution A while avoiding the formation of a white precipitate. The gel was placed in this solution and the bands usually appeared within five minutes; the development was complete in 15 minutes. After completion of the development, the gels were washed in 1% acetic acid for 20 minutes and dried.

**Table 1:** Silver staining of Agarose gels

1.	Acetate buffer	
a.	Sodium acetate	82 g/L
	Adjust pH to 6.0 using glacial acetic acid	
2.	Potassium ferrocyanide (2% w/v) in acetate buffer	
3.	Solution A	
	7% (w/v) sodium carbonate	
	Solution B	
	Ammonium nitrate	3 grams
	Silver nitrate	2 grams
	Silicotungstic acid	10 grams
	Formalin	8.8 mL
	Water to 1 L	

All solutions are stable at room temperature for at least 2 weeks, Solution B should be prepared by addition of ingredients in the order indicated

## Immunoblot analysis of CSF

Agarose isoelectric focusing was carried out as described above. Paired samples of CSF and serum (diluted 1:300) were run such that the IgG profile in the CSF could be compared to that of the serum. Application paper on which was loaded approximately 20 μL of CSF or diluted serum, was subjected to isoelectric focusing. Electrode solutions of 0.5 M acetic acid and 0.5 M sodium hydroxide were used as anodalyte and cathodalyte respectively. Focusing should be done in constant current mode for ideal results. The run was interrupted after the first 15 minutes (usually around 1,000 volts) to remove the strips. Additional focusing was carried out for 15 or 20 minutes until the voltage reached around 1,500 volts. The electrode strips were removed at the completion of the run and the gel was briefly washed in PBS. A suitably sized nitrocellulose paper soaked in PBS was used for the transfer of proteins from the gel to the nitrocellulose paper. Although we previously used PBS with 20% methanol to prepare the nitrocellulose for transfer, the use of methanol was not noted to add any advantage for the protein transfer and is no longer used. By simple contact and adsorption, all the proteins can be successfully transferred to the nitrocellulose paper after firm contact for 30 minutes. The gel was removed at the end of this period and the paper washed in PBS for five minutes for three changes. Quenching was accomplished by soaking the nitrocellulose in 5% horse serum for 15 minutes. After treatment of the gel for one hour with a suitable antiserum conjugated to alkaline phosphatase or peroxidase, the staining was completed using the appropriate substrate for color development. The nitrocellulose paper was dried and subjected to examination to detect oligoclonal bands that were present in the CSF but not in the serum. Known positive and negative samples were run in every sample to assure quality standards.

**Figure 1:**
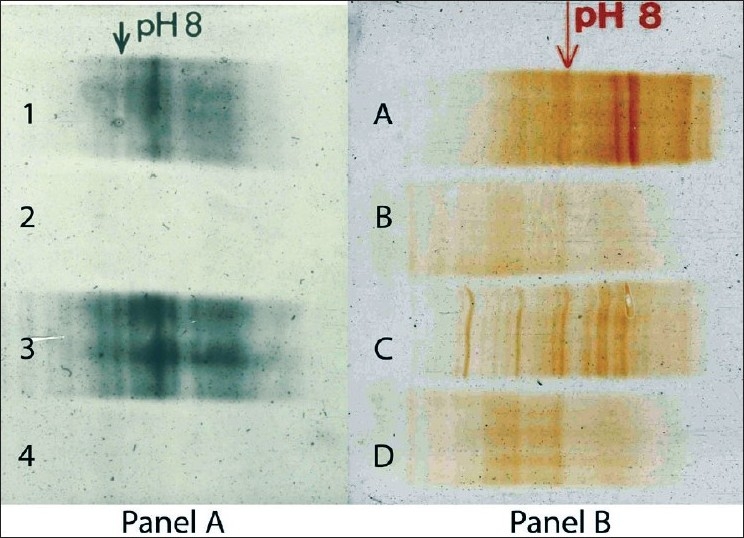
CSF from MS and nonMS subjects were examined by isoelectric focusing on Agarose gels. One half of the run was processed by silver staining (Panel A) and the other half by immunoblot analysis (Panel B). Lanes 1 and A, and lanes 3 and C represent CSF from 2 patients with MS and lanes 2 and B and 4 and D from 2 nonMS subjects. Note the improved resolution of the immunoblot gels

**Figure 2:**
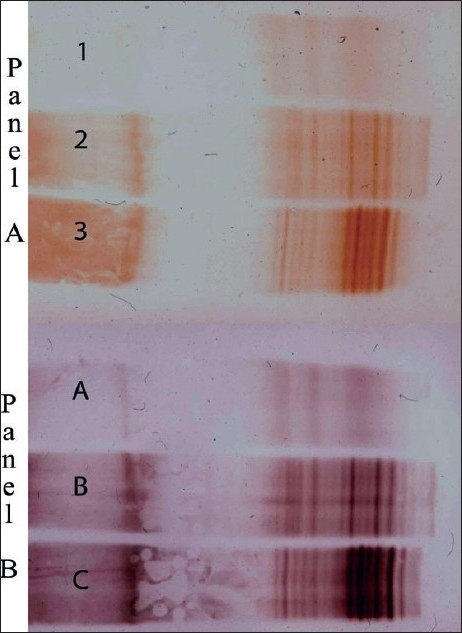
CSF from nonMS (Lanes 1 and A) and 2 MS patients (Lanes 2, 3 and B, C) were examined by immunoblot analysis. Unconcentrated CSF was used in Panel A, and CSF diluted 1/10 used in Panel B. DAB enhancement with silver (Panel B) permitted dilution of CSF for analysis with excellent resolution of the OCB bands

A comparison of the silver staining and immunoblot techniques was undertaken in identical CSF samples from MS and nonMS patients. Reproducibility and improved resolution were consistently observed with the immunoblot analysis [Figure 1]. Furthermore, the ability to do silver enhancement of diaminobenzidine permitted dilution of normal CSF to permit the detection of oligoclonal bands without any loss of resolution [Figure 2]. Today, we use unconcentrated CSF in isoelectric focusing studies for definition of oligoclonal bands and the carcinogenic potential of DAB has led to its replacement with 4-chloro-1-naphthol as a substrate for the development.

Presence of OCBs in the CSF is indicative of abnormally increased synthesis of intrathecal IgG. Often, but not always, this finding is associated with an elevation of IgG index and 24-hour IgG synthesis. While the latter two measures are quantitative, definition of IgG bands is a qualitative measure of the IgG produced, namely, an evaluation of the charge heterogeneity of the intrathecal immunoglobulins. Although quantitation of IgG bands can be quite readily accomplished, there is little value in this exercise as the number of bands seldom correlates with the degree of disability. Instead, it may be an indirect measure of the duration of the disease. Bands that appear seldom disappear; new bands appear over time and as the disease endures, the number of bands also increases. Although there is evidence that the number of bands seldom correlates with disability, there is some evidence to suggest that the disease is milder in band-negative patients, who comprise < 3% of all MS patients.[14–16]

Is CSF in early onset or childhood multiple sclerosis and acute disseminated encephalomyelitis (ADEM) different from that seen in adults? In a study that examined CSF from 136 patients with multiple sclerosis and disease onset before the age of 16 years, oligoclonal bands were seen in 92% of the patients.[17] Unlike in adults where CSF pleocytosis is uncommon even during an acute exacerbation, 66% of children had elevated CSF cell counts, and 30% had CSF cell counts > 30/cu.mm. In another study where ADEM was examined in pediatric patients, analysis of 54 spinal fluids did not identify bands in any patients.[18,19] Collectively, it would appear that the presence of OCBs would greatly favor a diagnosis of multiple sclerosis and its absence, a diagnosis of ADEM in the appropriate clinical setting. CSF from pediatric MS patients also had a higher incidence of IgM oligoclonal bands than that seen in adults.[17] The significance of this finding for future occurrence of progressive disease needs to be examined as the presence of IgM bands has been associated with the occurrence of a progressive course in adults with MS.[20–23]

In a recent study that examined OCBs in the CSF of 441 patients with MS and 77 patients with ADEM, OCBs unique to CSF were seen more consistently in MS (89%) but not in ADEM (10%).[24] Approximately 10% of MS patients and 84% of ADEM patients showed no bands or a “mirror pattern”, namely, identical bands in the serum and CSF. Interestingly, the CSF IEF pattern in experimental animals with autoimmune encephalomyelitis (EAE) also showed the mirror pattern, indicating that a systemic immune response predominates in EAE and ADEM. However, the immune response is predominantly intrathecal in MS. OCBs are therefore helpful in differentiating ADEM from MS as the presence of OCBs is more likely to be seen in MS than in ADEM.

Antigenic specificity of oligoclonal IgG has been the subject of intense investigations. Reactivity to antigens that are universal to MS patients but not to controls, is yet to be defined in CSF or serum. Reactivities of OCBs were examined using CSF from 14 MS and 14 normal individuals by using random hexamers expressed in a bacteriophage library.[25] The amino acid motif, RRPFF, was identified in the reactivities of IgG from 5/14 MS patients and 1/14 controls. Using a GenBank search, the sequence was identified in the Epstein Barr virus nuclear antigen (EBNA 1) and the heat-shock protein, α β crystalline, both antigens having been implicated in the pathogenesis of MS.[26]

Identification of OCBs is invaluable for the diagnosis of MS. According to the most recent McDonald criteria, if OCBs are present or an elevated IgG index is identified, this finding can be used with lesser stringent criteria on MRI to satisfy dissemination in space.[27] OCBs are also helpful as a predictor of the future occurrence of MS after a sentinel attack of demyelination compatible with the future occurrence of multiple sclerosis, namely, the Clinically Isolated Syndrome (CIS). Occurrence of OCBs in the CSF at presentation in a patient with CIS is a strong indicator of the future development of MS. In a study that examined MRI and CSF at baseline and during follow-up in 415 CIS patients followed over 50 months, the presence of OCBs in the CSF was observed to be a risk factor for progression to a second relapse and a diagnosis of clinical MS independent of abnormalities on the MRI. The presence of bands doubled the risk for developing MS in this population, but the presence or absence of OCBs did not seem to influence disability.[14]

## Proteomics of CSF

Two-dimensional electrophoresis followed by mass spectrometry of candidate “spots” resulted in the identification of numerous proteins in the human CSF. The technique termed “proteomics”, allows detailed separation of proteins in biological fluids. Analysis of the “proteome” of cells and body fluids offers promise to define pathogenesis of various disorders, and analysis of CSF from MS patients has been the focus of such attention. Detection of proteins unique for multiple sclerosis offered an opportunity to identify disease-specific proteins and opened up possibilities of identifying causative agents if such agents were expressed in the CSF. Pooled MS CSF from three patients and three inflammatory disorders of the CNS was examined in one such study.[28] CSF was concentrated 500 fold and examined for charge differences using isoelectric focusing in three separate pH gradients, and for molecular weight differences in the second dimension using SDS PAGE. A total of 430 spots were identified from 61 proteins in the MS CSF, of which all but four proteins were known to be present in normal human CSF. The four protein spots unique to MS CSF, however, failed to identify any protein that extended our understanding of this disorder (cartilage acidic protein, tetranectin, SPARC-like protein, and autotaxin t, a phosphodiasterase inhibitor). The roles of these four diverse proteins in the pathogenesis of MS, if any, remain to be defined.

In a subsequent study, MS CSF was examined using a different technique that did not require isoelectric focusing followed by SDS PAGE.[29] Instead, CSF was subjected to fractionation and concentration using ultrafiltration to enrich for proteins sized 5–50 KD. Proteins such as albumin, transferrin, and immunoglobulins that are large, abundant and interfered with the identification of smaller proteins, were thereby removed. The 5–50 KD retentate was subjected to pepsin digestion and two-dimensional liquid chromatography followed by gas phase fractionation in the ion trap. This gel-free approach identified 148 proteins in the lumbar CSF from MS patients and controls. Sixty new proteins not previously known to be in the CSF as well as a number of proteins unique to multiple sclerosis were identified. Some of these proteins show promise as biomarkers but quantitative studies in the CSF need to be performed to better define their roles in the pathogenesis of MS and to establish their respective roles as biomarkers for various functions of inflammation, demyelination, degeneration, and repair. Potential biomarkers and therapeutic targets for multiple sclerosis are presently being explored in other similar studies.[30,31]

It should be noted that potential biomarkers of MS can be proteins that are preferentially expressed in the CSF of MS patients, or can be proteins expressed in both MS and nonMS groups. The significance of proteins exclusively seen in the control but not the MS group is unclear. It should also be remembered that the techniques of proteomics are qualitative and not quantitative and do not differentiate proteins expressed preferentially in larger quantities in MS patients and therefore, probably bear significance from the standpoint of pathogenesis.

## Lipidomics in CSF

Phospholipids, ceramides, sphingomyelin, cerebrosides, cholesterol, and their derivatives constitute the bulk of the dry mass of the CNS. Analysis of lipids in the CSF from MS patients[32,33] did not identify disease-specific abnormalities, thus, interest in the study of lipids waned until recently. Electrospray ionization and atmospheric chemical spray ionization techniques have made it possible to examine lipids in the human CSF in a manner never previously possible.[34] Lipids serve as the basic scaffolding for neurons and glia and serve to insulate axons through lipid-laden myelin. They are a source of energy for cells and can be rapidly converted to signaling molecules and molecules that mediate inflammation through the production of prostaglandins and prostacyclines. Electrospray ionization studies identified 60 and 25% reduction of sulfatides in the plaque and in the adjacent normal-appearing white matter in the MS brain respectively.[35] Such studies in the CSF in MS will undoubtedly identify global lipid changes in MS that may provide valuable insights into diagnosis and pathogenesis of this disorder.

## Transcriptomics in the CSF

Transcriptomics is the study of “expression profiling” in the brain and consequently in the CSF, at a given point in time. Collection of the CSF under ideal conditions that maintain the integrity of mRNA is crucial in such studies as mRNA is very unstable. Once collected and appropriate cDNA molecules are produced, gene array technologies help to identify upregulated as well as downregulated genes. This fledgling technology is currently in a state of development for CSF and may serve to define changes that characterize the various stages of multiple sclerosis, inflammation, demyelination, remyelination, and neural genesis. In a recent study, the transcriptome from the B cells in CSF in MS was correlated to the proteome identified by mass spectrometry. Complete correspondence was identified between the V(D)J recombination and somatic hypermutation of the variable region of the immunoglobulin gene and sequences in the variable region of the IgG, indicating that B cells are indeed the source of some of the components of the proteome seen in the CSF in MS patients.[36]

## Biomarkers in CSF

### Inflammation in MS

Soluble mediators of inflammation, namely, lymphokines, prostaglandins, chemokines, and C5b-9 complement membrane attack complex have been examined in CSF in limited studies of multiple sclerosis.[37–39] IL1, IL12, TNF-α, INF, IL10, TGF-β, and adhesion molecules and their soluble receptors as well as MMP 9 all examined states of inflammation or quiescence.[40–43] A modest correlation was identified between some of these inflammatory markers in serum or CSF and evidence of disease activity on MRI.[44–47] None have lived up to the expectation of a surrogate for clinical disease activity or progression.

### Markers of Demyelination

How well does the CSF reflect demyelination in the CNS? Pioneering studies of electron microscopy of sediments from ultracentrifuged CSF from MS patients identified the typical multilamellar myelin fragments with distinct major dense line and interperiod lines as extracellular fragments during clinical exacerbations.[48] These observations led to the examination of myelin proteins in CSF as markers of demyelination. “MBP-like material” (MBPL) is probably the best studied; immunoreactive MBPL or its fragments can be detected in the CSF during acute exacerbations in 80% of remitting-relapsing MS patients undergoing acute exacerbations.[49–51] CSF MBPL levels rise acutely during the exacerbation and subside over the next 4–6 weeks. However, evaluation of MBPL has not lived up to the expectation of a surrogate for demyelination in the CSF for a number of reasons. MBP levels in the lumbar CSF may be normal in acute optic neuritis[52] but are often abnormal in patients undergoing active myelitis.[53,54] This is probably just a reflection of anatomical proximity or distance of the sampling site to the location of demyelination. Immunoreactive MBPL or its fragments can also be detected in the CSF in disorders other than multiple sclerosis, and its presence is merely a marker of myelin injury, be it multiple sclerosis, trauma, cerebrovascular accident, or any disorder that can cause myelin injury. However, it should be noted that MBP fragment 43–88 was notably absent in the CSF in one study in 8/10 patients with ADEM a disorder with disseminated white matter injury,.[55] It has been suggested that detection of specific MBP fragments may be more specific for MS exacerbations as the size of MBP seen in the CSF of patients with cerebrovascular events is considerably different from the fragments observed from CSF of patients with MS. Fragments of MBP are generated by degradation of MBP by lysozomal carboxyendo peptidases, especially cathepsin D, which is commonly present at sites of multiple sclerosis demyelination.[56] The usefulness of detection of specific fragments that confer specificity of detection of MBP-like material to multiple sclerosis is yet to be demonstrated.

Is there any clinical utility in the measurement of CSF MBP? Measurement of CSF MBP levels in multiple sclerosis is sometimes used to confirm a clinical exacerbation as elevated levels are generally not seen in patients during periods of remission. Elevated levels usually remain elevated for 4–6 weeks and therefore can be used as a marker for recent disease activity. A normal value however does not imply disease quiescence for the reasons identified above. However, it should be noted that the definition of disease activity used in most of these studies predates the MRI era of investigations in MS, and therefore does not reflect true disease activity as defined by gadolinium-enhancing lesions. The limitations outweigh the usefulness of evaluating MBP levels in the CSF due to which measurement of CSF MBP levels is not the standard of care in routine analysis of CSF of patients with multiple sclerosis.

### Absence of Markers for Remyelination

Remyelination is not the primary mechanism of recovery of function in multiple sclerosis. In fact, most lesions remain demyelinated at the time when return of function occurs. This is best seen in optic neuritis where the optic nerves, which are commonly affected, show severe demyelination at the site of the original optic neuritis at autopsy, although during life, the patient reported normal or near-normal vision. Often, this is the sentinel event that led to the future occurrence of MS, an event that may have occurred some 30–50 years prior to death. Many patients have trouble remembering which eye was affected during the sentinel optic neuritis although return of myelin did not occur. In the nonhuman primate model of demyelination with lysolecithin, remyelination was found to be slow in the optic nerve as compared to the spinal cord.[57] Although “shadow plaques” occur in multiple sclerosis, remyelination is the exception rather than the rule in the evolution of most lesions of multiple sclerosis. There are no good markers for remyelination that allow the detection of shadow plaques in patients during life. Voxel-based magnetization transfer imaging attempts to define parameters for remyelination in MS, but the utility of this technique has remained a research tool.[58] This inability to detect remyelination has hampered the identification of markers in the CSF that correlate with remyelination. At this time, there are no markers for detection of remyelination in the CSF.

### Markers of progression and disability

Cellular and molecular substrates of progression and disability in MS are only beginning to be understood. Axonal injury is without doubt a significant contributor to disability as also astrogliosis of the demyelinated plaque, which may impede repair and regeneration. There are several markers in the CSF that reflect these changes and show promise as surrogate markers in the CSF for injury that correlate with disability and progression.

Phosphorylated forms of neurofilament proteins maintain the integrity of the axon and their repulsion of negative charges keep the axons bulky and cylindrical. With axonal injury, the neurofilaments become nonphosphorylated and the axons collapse and can no longer sustain normal function. Antibodies to nonphosphorylated neurofilaments have been useful in detection of injured axons in confocal microscopy. Recently, these antibodies have been used to detect the extent of axonal injury characterized by the presence of nonphosphorylated neurofilaments in the CSF.[59] The study identified that elevated CSF levels of neurofilament protein predict, as an independent variable, the likelihood of development of future multiple sclerosis in patients with CIS.

Three groups of proteins constitute normal neurofilaments; light, medium, and heavy chains. A number of studies have identified that neurofilament light chains are increased in the CSF in the majority of patients with relapsing, remitting multiple sclerosis.[60–62] There is some preliminary evidence that neurofilament heavy chain levels may be raised in the CSF in patients with optic neuritis or multiple sclerosis, and that such levels may be a predictor of the presence of enhancing lesions on MRI as well as elevated levels of MBP in the CSF. Of interest was the observation that the change in CSF neurofilament heavy chain from baseline to three weeks was a predictor of the subsequent clinical outcome in the MS but not of the optic neuritis group.[63] Of interest is also the observation that antibodies to neurofilaments occur in the serum and CSF of patients with MS. Such antibodies were identified in relapsing as well as progressive disease and may serve as markers of progressive axonal injury. As to whether these antibodies have a role in the pathogenesis of the relapsing and progressive forms of multiple sclerosis needs to be established. If serum titers to neurofilament light chain are significantly correlated with axonal injury, this antibody measurement in serum can be an index of axonal injury in MS and serve as a suitable surrogate for disease progression.

Glial fibrillary acid protein (GFAP) and protein S100B are proteins that are unique to the glia. GFAP, the major protein of the glial intermediate filament, was first isolated from an MS plaque from the fibrous astrocytes that densely populate a typical plaque.[64] Protein S100B is a cytosolic protein found in astrocytes and oligodendrocytes. Gliosis is the rule rather than the exception in the organization of an MS plaque, and therefore, GFAP and protein S-100B were examined in the CSF in patients with MS.[62] CSF levels of GFAP and S100B were elevated in patients with relapsing as well as progressive disease. Both these proteins may serve as markers of glial proliferation in areas of demyelination and axonal injury.[63,65] As protein S100B is also expressed by oligodendrocytes, it is possible that its levels in the CSF could reflect not only astrogliosis but also remyelination. Collectively, they may also serve as markers of disease progression. Longitudinal analysis of these proteins in the CSF needs to be done and their utility in diagnosis and prognosis regarding disability and progression established.

In conclusion, CSF evaluation permits the examination of changes in the CNS that occurs in health and disease. With the advent of refined techniques that generate large amounts of data and better storage and analysis of data using bioinformatics, information is becoming available from CSF that aid in the diagnosis, treatment, and research in multiple sclerosis. Advances from these areas will surely change the face of multiple sclerosis and empower the patient and physician to understand and treat this disorder better in the years ahead.
